# Beetle elytra: evolution, modifications and biological functions

**DOI:** 10.1098/rsbl.2022.0559

**Published:** 2023-03-01

**Authors:** Jakub Goczał, Rolf G. Beutel

**Affiliations:** ^1^ Department of Forest Ecosystems Protection, University of Agriculture in Krakow, 29 Listopada 54, 31-425 Krakow, Poland; ^2^ Friedrich-Schiller-Universität Jena, Institut für Zoologie und Evolutionsforschung, 07743 Jena, Germany

**Keywords:** forewing, evolution, development, Coleoptera, morphology, biomaterial

## Abstract

Conversion of forewings into hardened covers, elytra, was a ground-breaking morphological adaptation that has contributed to the extraordinary evolutionary success of beetles. Nevertheless, the knowledge of the functional aspects of these structures is still fragmentary and scattered across a large number of studies. Here, we have synthesized the presently available information on the evolution, development, modifications and biological functions of this crucial evolutionary novelty. The formation of elytra took place in the earliest evolution of Coleoptera, very likely already in the Carboniferous, and was achieved through the gradual process of progressive forewing sclerotization and the formation of inward directed epipleura and a secluded sub-elytral space. In many lineages of modern beetles, the elytra have been distinctly modified. This includes multiple surface modifications, a rigid connection or fusion of the elytra, or partial or complete reduction. Beetle elytra can be involved in a very broad spectrum of functions: mechanical protection of hind wings and body, anti-predator strategies, thermoregulation and water saving, water harvesting, flight, hind wing folding, diving and swimming, self-cleaning and burrow cleaning, phoresy of symbiotic organisms, mating and courtship, and acoustic communication. We postulate that the potential of the elytra to take over multiple tasks has enormously contributed to the unparalleled diversification of beetles.

## 1. Introduction

About a quarter of all known extant animal species on Earth belong to one particular order—Coleoptera (beetles) [1,2]. The extraordinary evolutionary success of this group is considered to be linked to a series of evolutionary events and specific traits, including the co-radiation with angiosperm plants in the Cretaceous [3–5], long survival of lineages and sustained diversification in a variety of niches [2], and also to several evolutionary novelties [1,6–8]. Among the morphological innovations, one particular evolutionary transformation stands out—the conversion of the forewings into hardened, protective covers called elytra. This is considered as a crucial morphological adaptation that contributed to the evolutionary success of the megadiverse order [1,6,8–10]. However, the transformation of forewings into hard covers also took place in several hemimetabolous groups of moderate diversity, including for instance the extant order Dermaptera (e.g. [11]) or the extinct dictyopteran †Umenocoleidae [12].

Elytra are commonly perceived as an evolutionary key innovation of Coleoptera. Nevertheless, their evolutionary aspects, various modifications and especially biological functions are still incompletely understood, and relevant information is widely scattered across many studies. Consequently, the main goal of the present review is to synthesize the current state of knowledge on the evolution, modifications and possible role of elytra in different functional contexts. This is crucial for a deeper understanding of the role of elytra in the unparalleled diversification of beetles.

## 2. Evolution and development of beetle elytra

**Figure 1.**
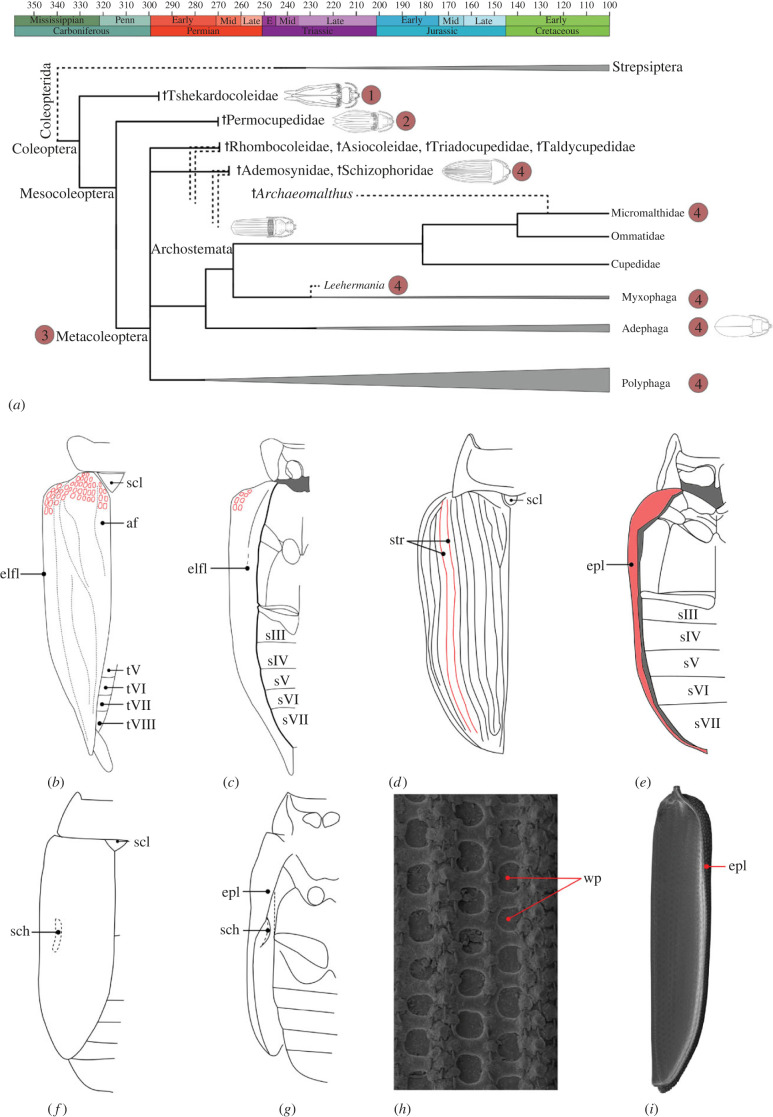
(*a*) Hypothesis for the early evolution of Coleoptera (and elytra), based on [14]. Numbers (1–4) indicate crucial steps in the evolution of elytra: (1) elytra distinctly surpassing the abdominal apex, lacking epipleura folded inwards, not tightly fitting with the abdomen, with partially maintained venation and window punctures—e.g. †Tshekardocoleidae; (2) elytra slightly surpassing the abdominal apex, with a parallel arrangement of longitudinal veins—†Permocupedidae; (3) elytra not surpassing the abdominal apex, tightly fitting with the abdomen, forming a secluded sub-elytral space—e.g. †Rhombocoleidae, †Taldycupedidae, all extant beetles (with few secondary exceptions); (4) elytra tightly fitting, without window punctures, smooth or with striae (or other surface patterns). Age estimates of nodes are approximations (see [14]). Schematic drawings from [14]. (*b,c*) Reconstruction of postcephalic body of a Lower Permian beetle (†Tshekardocoleidae/†*Coleopsis*), based on [14,15]: (*b*) dorsal view; (*c*) ventral view (note that window punctures (in red) are present on entire elytral surface). (*d,e*) Postcephalic body of †*Peltosyne triassica* Ponomarenko (†Peltosynidae), modified from [16]: (*d*) dorsal view, (*e*) ventral view. (*f,g*) Postcephalic body of †*Abrhadeocoleodes ooidus* Tan, Ren, Shih & Yang (†Schizophoridae), modified from [17]: (*f*) dorsal view, (*g*) ventral view (elytron slightly extended, displaying schiza). (*h,i*) details of *Tetraphalerus bruchi* Heller (Archostemata, Ommatidae) (SEM micrographs courtesy of F. Friedrich): (*h*) elytral window punctures, (*i*) entire elytron, epipleuron and inner surface. Abbreviations: elfl—flat lateral elytral flange, epl—infolded epipleura, str—longitudinal elytral striae, sch—schiza (ridge) of internal elytral surface, scl—mesoscutellar shield (locking device), wp—window punctures, af—anal field, t(VIII -V)—abdominal tergites, s(VII-III)—abdominal sternites.

Elytra are not only the most conspicuous feature of beetles, but also a fundamental evolutionary novelty. Sclerotized forewings very likely evolved in the Late Carboniferous [11,13] (figure 1*a*). One of the most widely discussed drivers of the formation of protective wing cases is a preference for specific wood-associated microhabitats, especially below bark [11]. Characteristic features such as window punctures (small areas of thin, semi-transparent cuticle) (figure 1*b,c,h,i*) and cuticular tubercles (and possibly scales) on the elytral surface (and other body regions) can be found in different extinct lineages, but also in wood-associated species of two extant families of the relictual suborder Archostemata [18,19] (figure 1*a*). Based on this similarity, it appears plausible that more or less rigid elytra enabled early beetles to inhabit subcortical microhabitats (narrow spaces below bark), as it applies to species of the extant archostematan families Ommatidae and Cupedidae [20]. Inhabiting subcortical spaces provides advantages, especially reducing competition and predation pressure, as well as ensuring appropriate humidity and reduced water loss. Like extant Archostemata, early stem group beetles very likely lacked cryptonephric Malpighian tubules (e.g. [6]), which are present in modern beetles of the megadiverse polyphagan Cucujiformia. These specialized excretory organs enhance water re-absorption, and thus allow beetles to live in drier conditions, especially exposed on plant surfaces.

Elytra of early Permian stem group beetles (Coleoptera *sensu lato*), †Tshekardocoleidae and †*Coleopsis* [14,15], distinctly surpassed the elytral apex posteriorly, lacked epipleura folded inwards, and did not fit tightly with the abdomen, thus only loosely covering the posterior body [14,15] (figure 1*a–c*). Moreover, they had partially maintained the original longitudinal wing venation (figure 1*b*), lacking the parallel arrangement of later extinct groups and modern beetles [15,21–23] (figure 1*d*).

The later branches of stem group beetles were characterized by a parallel arrangement of semi-sclerotized elytral cells (e.g. †Permocupedidae, †Rhombocoleidae; [13,18,22,24]. Elytra lacking these window punctures are found in all extant beetles except for the small families Cupedidae and Ommatidae, and evolved already in the Permian (e.g. [19,25] (figure 1*a*). However, these transformations, which included evenly sclerotized elytra, took place several times, also in the few extant archostematan species not belonging to Ommatidae and Cupedidae [14].

The early transformations of the forewings suggest that the progressive sclerotization of veins was likely a first step towards the formation of evenly sclerotized elytra, lacking the small zones of weakness (window punctures) (figure 1*h*). This is in agreement with experimental findings based on an ‘evo-devo’ approach [8]. A smooth and firm surface lacking vestiges of the original venation occurs in several extinct groups from the Late Permian to the Early to Mid-Mesozoic (e.g. †Peltosynidae, †Ademosynidae, †Schizophoridae; [16,18,20] (figure 1*d,e*) and is present in almost all modern beetles (crown group, Coleoptera *sensu stricto*) [26].

The earliest known elytra [15,23] already differ distinctly from membranous wings suitable for flight, lacking a large anal field (figure 1*b*), and displaying a straight posterior edge and a pointed apex (e.g. [18]; figure 1*b,c*). A crucial transformation was the formation of epipleura folded inwards (figure 1*e,g,i*), instead of broad and flat lateral flanges [14,15] (figure 1*b,c*), and an adjustment of the length and width to the shape of the metathorax and abdomen. An intermediate condition was present in †Permocupedidae [14,18,27,28], but all following groups (Metacoleoptera) [14] are characterized by a close fit of elytra and posterior body, and consequently a secluded sub-elytral space. Moreover, an entire series of locking devices (see [14,29]) evolved (e.g. mesoscutellar shield; figure 1*b,d,f*), ensuring a firm connection of the right and left elytra in the resting position, and thus enhancing the protective function of the elytra.

Specialized structures that appeared early in the evolution of beetles are schiza, protrusions of the internal side of the elytra (figure 1*f,g*). These structures, probably an additional locking device (e.g. [14,17,18]), occur in †Schizophoridae (figure 1*f,g*) and many †Rhombocoleidae [14,18,30]. Claims that their presence is related to aquatic habits, combined with smooth elytra in †Schizophoridae (see [30]), requires confirmation.

The formation of elytra occurred together with other modifications, including longitudinal and transverse hind wing folding which were explored by Haas [31] (see also [32]), enlargement of the metathorax, simplification of the pterothoracic musculature [33] and formation of elytral sensory structures [6,34].

The developmental mechanism that resulted in the formation of elytra remained unexplored until recently [8,35]. A series of ‘evo-devo’ studies [8,35–37] showed that the elytra most likely evolved through a gradual process of forewing ‘exoskeletalization’ [35], which was achieved through the co-option of genes regulating increased cuticle hardening of other body parts and their integration into the wing gene network [8,35,37]. The co-option took place in at least three stages: dorsal ‘exoskeletalization’ regulated by the apterous genes (*ap*), pre-vein ‘exoskeletalization’ (genetic modulator unknown), and the ‘exoskeletalization’ of adjacent sensory bristles (modulated by the single beetle homologue of *ac* and *sc*). A recent study based on the exploration of wing transcriptomes has uncovered novel genes involved in the process of elytra formation, especially in hardening and pigmentation—*Tc-hr38* (TC013146), formation of elytral sensory structures—*Tc-hr38* (TC013146) and formation of elytral venation—*Tc-chemosensory10* (TC008682) [34]. Shape transition resulting in closely fitting of wing cases was achieved through the neo-functionalization of the wing gene, *abrupt* [38]. A study based on model species has also shown that two main structural proteins, TcCPR18 and TcCPR27, are crucial during the process of elytral hardening [39]. It is also likely that the formation of elytra and the corresponding higher investment of cuticle has resulted in increased demands for the semi-essential amino acid tyrosine. This has been solved in several beetle lineages by associating with symbiotic microorganisms [40–42].

## 3. Elytra modifications

### (a) Elytral shortening or loss

The vast majority of beetles (except e.g. Staphylinoidea) are characterized by well-developed elytra that fully cover the abdomen. However, there are few examples of completely apterous species (lacking metathoracic wings and elytra), for instance neotenic females of many fireflies (Lampyridae). A relatively frequent phenomenon is elytral shortening, which can be found for instance in glow-worms (Phengodidae) or in several groups of Cerambycidae (e.g. Necydalinae) (see [43]). The reduction has occurred with different intensity multiple times in the evolutionary history of beetles [43–46]. Although the selective benefit of brachelytrism is still unclear, most current hypotheses refer to mimicry, energy saving, or increased manoeuvrability as potential drivers of elytral reduction [43,46,47]. Recent studies indicate that elytral shortening/secondary elongation might have occurred repeatedly in certain beetle lineages [46,48].

### (b) Elytral fusion

Another elytral modification is the fusion along the mesal edge, which occurs relatively frequently in flightless epigean beetles, or in species burrowing in soil. Examples are representatives of Carabidae (e.g. in *Carabus* Linnaeus), Chrysomelidae, Zopherinae, Tenebrionidae or Geotrupidae, especially species living arid environments [49,50]. A well-known case of exposed and phytophagous beetles with elytral fusion are Cetoniinae (Scarabaeoidea), where a recess of the epipleural region allows extension and movements of the hind wings with closed elytra (e.g. [33]). A widely discussed driver of elytral fusion is selective pressure to minimize water loss [50]. It is also conceivable that fusion was caused by selective pressure on an increase of mechanical protection, which might be essential to withstand specific types of predation and other external stressors [51]. Recent research on ‘non-crushable’ ironclad beetles (Zopherinae) revealed unique modifications. An extraordinary design of interlocking sutures allows these beetles to withstand an extreme load force before fracturing [52].

### (c) Elytral surface modifications

Elytra underwent manifold structural modifications, deviating in multiple ways from the ancestral state (figure 1*b,c*). Wing cases of extant beetles exhibit extreme variation in shape, sculpture and armature (e.g. spines, tubercles and denticles), and also regarding the vestiture of setae or microtrichia, even among closely related taxa. Different forms of longitudinal rows of surface punctures, setae or microtrichia are also common (e.g. [11]). Various armatures of an elytral declivity are characteristic for bark beetles (Curculionidae: Scolytinae) [53]. Solid elytral spines or spikes have evolved several times and occur for instance in leaf beetles of Hispinae [54], in the pleasing fungus beetle *Ellipticus spinifer* (Thomson) (Erotylidae), in *Cacodaemon* Thomson and *Amphisternus* Germar of Endomychidae, or within weevils, for instance in *Hoplapoderus* Jekel (Attelabidae) and *Catasarcus* Schönherr (Curculionidae). Another type of elytral modification is widening beyond the body margin, which results in a turtle-like appearance in leaf beetles of Cassidinae, darkling beetles (Tenebrionidae) (e.g. Cossyphodini) and several genera of Trogossitidae (e.g. *Trichocateres* Kolibáč). An extreme example of elytral expansion occurs in the tropical violin beetle *Mormolyce phyllodes* Hagenbach (Carabidae).

The arrangement of the elytra is apparently driven by various forms of selective pressure. The function of some modifications is discussed in the following section—‘Functions of elytra’.

## 4. Functions of elytra

### (a) Protection and defence

**Figure 2.**
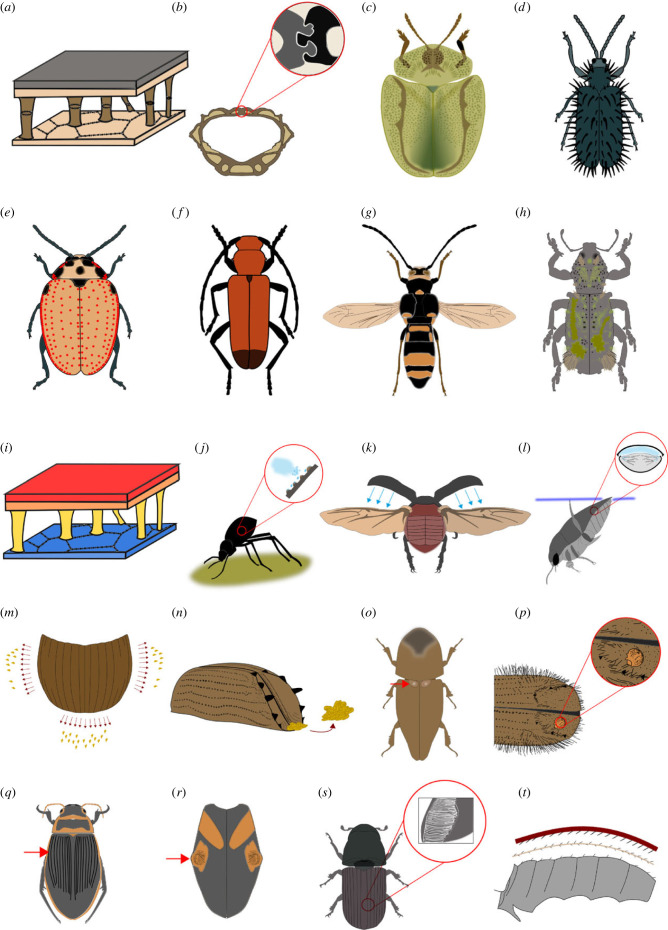
(*a–h*) The role of elytra in protection and defence. (*a*) Internal elytral structure (simplified model) provides high mechanical resistance; (*b*) toughening mechanisms of the elytra of *Nosoderma diabolicum* (LeConte) (Zopheridae) [51]; (*c*) widened elytra in Cassidinae (Chrysomelidae); (*d*) defensive spikes of *Hispa* Linnaeus spp. (Chrysomelidae); (*e*) defensive glands on elytra and pronotum of *Diamphidia nigroornata* (Stål) (Chrysomelidae) [55]; (*f*) red elytral warning coloration of *Euryphagus lundii* (Fabricius) (Cerambycidae); (*g*) elytra shortening as part of mimicry in *Hesthesis variegate* (Fabricius) (Cerambycidae); (*h*) epizootic moss garden on the elytra surface of *Lithinus rufopenicillatus* Fairmaire (Curculionidae)—camouflage mechanism. (*i–t*) Examples of additional (not related to protection) functions of elytra: (*i*) internal structure results in thermal isolation and reduced water loss; (*j*) specific microstructures allow for efficient fog-basking; (*k*) elytra provide lift and support balance during flight; (*l*) air stored in the sub-elytral chamber is used for respiration by aquatic beetles and surface properties affect the hydrodynamics of swimming; (*m*) elytral anti-adhesive properties facilitate burrowing in sticky mediums—visualization of the repulsion of soil particles; (*n*) the elytral declivity of bark beetles functions as a shovel to remove faeces and detritus from the gallery; (*o*) elytral mycangia are used for safe transport of symbiotic fungi; (*p*) small organisms can be transported on or below elytra—visualization of a mite attached to the elytral declivity of a bark beetle; (*q*) furrows on the elytral surface of female *Dytiscus* Linnaeus (Dytiscidae) weaken the male's grip and increase the female's control on mating. (*r*) Elytral glandular notches of *Clavicollis fugiens* Marseul (Anthicidae) are used for storage of cantharidin, which is transferred to the female as a nuptial gift [56]. (*s*) Elytra as an integral part of different types of stridulatory devices—visualization of a stridulatory device in a bark beetle; (*t*) microtrichia on the inner elytral surface play a role in hind wing folding and locking.

Elytra owe their protective properties to mechanical interactions between internal layers and their sublayers (figure 2*a*), and also to different arrangements of polysaccharide–protein fibres within each sublayer [57]. However, empirical studies testing the elytral protective function are dramatically scarce. Only a single study provides data on the elytral role in hind wing protection [58]. A group of *Tribolium castaneum* (Herbts) (Tenebrionidae) individuals with surgically removed elytra experienced significantly more damage to their membranous alae during predator attacks than a control group with intact wing cases [58]. The mortality level due to attacks from a wolf spider was clearly higher in the group without elytra [58]. It was also shown that selective pressure on maximizing elytral mechanical resistance to specific types of predation and other high external loads can result in distinct modifications of the internal elytral structure and interlocking mechanism [51,54] (figure 2*b*).

Not only the internal configuration, but also external structures (e.g. spines, tubercles, denticles) likely play a role in anti-predation strategies. Widening of the elytra margins or formation of prominent dorsal spines in some leaf beetles (figure 2*c,d*) can be linked to adaptations to specific predatory regimes [59]. Specifically shaped denticles on the elytral declivity of bark beetles were also interpreted as protective structures used in burrow-blocking against predators and burrow-usurpers [60].

It is noteworthy that the mechanical protective function is significantly reduced or even absent in some groups. The vestigial elytra in Atractocerinae (Lymexylidae) or Necydalinae (Cerambycidae) can neither protect the integument nor internal organs of the abdomen or the exposed hind wings. There are also soft-bodied beetles (e.g. many Melyridae or Cantharidae) with a distinctly reduced degree of sclerotization, including relatively thin and soft elytra, which can be pierced comparatively easily. Various alternative defence strategies have been identified in groups of beetles with reduced mechanical protection provided by elytra, including chemical protection, mimicry, hardening of the abdominal tergites or aposematic coloration [6,61,62]. It is very likely that the mentioned alternative defence strategies might compensate for the limited protective role of the elytra in these groups [62].

In some cases, chemical defence resulted in the formation of specific elytral structures. Glands producing toxic secretions occur on the elytral surface of many leaf beetles [55] (figure 2*e*). A lateral elytral flange of ground beetles of Paussinae is used for well-aimed spraying of explosive defensive substances [63].

Elytral coloration plays an important role in anti-predator strategies. An aposematic (warning) bright coloration of elytra can be found in numerous groups representing distantly related lineages (e.g. within Chrysomeloidea or Cleroidea figure 2*f*). Nevertheless, few studies have empirically established how different elytral coloration and patterning affects predation pressure. It has been shown that orange-black elytral colouring of burying beetles of species of *Nicrophorus* Fabricius (Silphidae) plays an aposematic function against avian predators [64]. Another empirical study on ladybirds has revealed that beetles with spotted elytra were attacked less frequently by birds, and that the removal of elytra significantly increases the number of attacks [65]. It was also shown that the intensity of the elytral warning coloration (e.g. red) might be directly related to the toxic alkaloid content in lady beetles. This suggests that the coloration might serve as a distinct warning signal for potential predators that rely on visual cues [66].

Characteristic coloration, patterning or even distinct structural modifications might be attributed to mimicry strategies. A large number of diurnal herbivorous beetles mimic wasps, displaying black-yellow or black-orange elytral patterns. Elytra and other body parts of many scarab beetles of Glaphyridae bear a dense vestiture of long hairs, which makes them resemble bumblebees. The elytra of the longhorn beetle *Hesthesis variegata* (Fabricius) are largely reduced, thus exposing narrowed hind wings (figure 2*g*). Moreover, the abdomen displays a pattern of black and yellow stripes, which makes these beetles look very similar to wasps of the subfamily Eumeninae (figure 2*g*).

Elytra are often involved in camouflage mechanisms. Cryptic coloration characterizes numerous species, frequently combined with distinct structural modification enhancing the camouflage effect. A unique combination of elytral indentations, crests and tubercles of *Pristoderus chloreus* Turco & Ślipiński (Zopheridae) [67] makes it look like a lump of lichen on bark. The rough, hairy elytra surface of the weevil *Lithinus rufopenicillatus* (Fairmaire) allows it to cultivate an epizootic moss garden on its body, providing excellent camouflage (figure 2*h*). The prominent spine on the central elytral region of leaf beetles of Dorynotini appears like a thorn of the host plant, which is likely an efficient camouflage mechanism [68].

Apart from predation, another potential risk is falling from a great height during flight or from a tree or other elevated locations. It was shown that elytra can absorb a significant part of the collision energy, protecting internal organs against damage [69]. This can be attributed to the internal elytral structure, notably to columnar trabecular structures [57] (figure 2*a*). Elytra coupling mechanisms can also play a role, as unlocking during the fall can significantly increase the impact energy absorption [69].

### (b) Thermoregulation

Elytra distinctly contribute to the passive thermoregulation of the body temperature, playing a double role in this process. The first function is either to absorb a fraction of the radiation to increase the body temperature, or alternatively to reflect it to prevent overheating (figure 2*i*). Several elytral characteristics including coloration and patterning, thickness or surface microstructure (e.g. presence of specific scales) likely have a distinct effect on absorption/reflection properties [70,71]. A recent empirical study on *T. castaneum* showed that elytra are also critical for withstanding a brief cold-shock [58]. The second role of elytra in body thermoregulation is closely related to another function, the reduction of water loss (see Water saving section). Saved water resources can be used in body thermoregulation through evaporative cooling mechanisms [49,72,73].

### (c) Water saving

The ability to conserve water is fundamental for cellular functioning in terrestrial animals, and to solve this problem was crucial in the context of terrestrialization [74]. The formation of elytra has greatly improved the efficiency of water conservation. Elytra provide a mechanical barrier for direct water loss from the body surface by covering two critical areas: the thin dorsal integument of the abdomen and the spiracles, which have been recognized as main places of water loss [75]. It was documented that the desiccation tolerance of beetles with removed elytra is significantly lowered when compared to a control group with intact elytra [58]. Different morphological adjustments can help to reduce water loss. In some arid-adapted beetles (e.g. several groups of Tenebrionidae), highly specialized dermal glands were identified on the elytra. They produce wax bloom, an admixture of lipids, proteins and pigments, that reduces evaporative water loss [76]. Another adaptation in this context is elytral fusion, along with the formation of a tightly secluded sub-elytral chamber. This configuration results in a thermal buffer and serves as a respiratory water-saving device [58,77,78].

### (d) Water harvesting

A remarkable elytral function occurs in species of darkling beetles inhabiting the Namib Desert. Some use their specialized elytra for water capture by fog-basking (figure 2*j*). Standing on sand dune ridges in a characteristic posture they obtain water through increased fog condensation on hydrophilic peaks of elytral tubercles, which are surrounded by hydrophobic areas [79]. Although the elytral surface microstructure has a significant effect on water harvesting effectiveness, the behavioural aspects (e.g. adopting a suitable posture) were also found to be important in this process [79].

### (e) Flight

The active role of the fore wings in flight is more or less completely obsolete in the vast majority of extant beetles. A notable exception is the archostematan species *Priacma serrata* LeConte, where the elytra are still actively moved during flight [80], and thus contribute to the propulsive force.

In the vast majority of beetles, the elytra are extended laterally during flight (figure 2*k*). However, in some groups, they remain linked or are scarcely opened (Cetoniinae or genus *Scarabaeus* Linnaeus (Scarabaeidae)). In almost all living beetle species, the elytra movements are driven passively through the mechanical coupling between the meta- and mesothorax [29]. However, Sitorus *et al.* [81] suggested that elytra of certain species can compensate their own weight by producing additional aerodynamic force (figure 2*k*). A recent detailed study with *Trypoxylus dichotomus* (Linnaeus) (Scarabaeidae) showed that without interactions with the hind wings, the vertical force generated by elytra is too small to compensate their own weight [82]. However, due to interaction between both wing pairs, the vertical force generated by the elytra could be increased by up to 80%, fully compensating the elytral weight and increasing the vertical force of the hind wing by about 6% [82]. Laterally extended elytra also generate passive aerodynamic stabilization during flight [83]. In the miniature featherwing beetles (Ptiliidae), the elytra act as ‘inertial brakes’, preventing undue body oscillation during their specific mode of flight [84]. The elytra are equipped with specialized campaniform sensilla, providing feedback required for flight control [85,86].

### (f) Diving and swimming

The formation of a secluded sub-elytral chamber was a prerequisite for multiple invasions of the aquatic environment in Coleoptera [6,27]. It was found that both elytral shape and surface texture have a significant effect on hydrokinetics of water beetles and reflect adaptations to different modes of swimming [87,88]. An air bubble obtained from the surface and stored in the sub-elytral chamber of many aquatic beetles, especially in Adephaga, is used for respiration when diving (figure 2*l*). It also generates buoyancy, preventing the beetles from sinking to the ground and facilitates returning to the surface to renew the oxygen supply. Moreover, it was found that in whirligig beetles (Gyrinidae) inboard abduction of the elytra is crucial for manoeuvrability, especially for turning, as they increase drag and act as a pivot during this movement [89].

### (g) Self-cleaning and burrow cleaning

Anti-adhesive properties of the elytra surface (figure 2*m*) were discovered in several groups of soil-burrowing scarab beetles, and it was shown that they help to burrow into sticky soil and also dung [90]. A recent study indicated that these unique properties of beetle wing cases are due to the molecular polarity of the elytral surface [91]. Different burrow cleaning strategies evolved in several groups of bark beetles, leading to the formation of a unique shovel-like elytral declivity [60]. In some species of these eusocial beetles, one or rarely both parents keep burrows clean by removing faeces and detritus using the declivity as a shovel [60] (figure 2*n*).

### (h) Phoresy

Effective transport of symbiotic organisms (e.g. fungi) is crucial for microbial mutualists. This has led to the formation of different structural configurations for storing microsymbionts during dispersal. Specific external exoskeletal cavities called mycangia were identified in many groups [92]. They are used for the transport of symbiotic fungi [92,93]. Exoskeletal cavities can be located on different body parts, including the elytra [92]. Elytral mycangia were observed for example in various ambrosia beetles of Xyleborini (Scolytinae and Platypodinae, Curculionidae) [93] (figure 2*o*).

Other small organisms, both mutualistic and parasitic, are also frequently associated with beetle elytra, for instance mites and nematodes. In many cases, they are transported in the sub-elytral cavity or, in the case of some nematodes, in pocket-like structures (nematangia) [94] of beetle hind wings. By contrast, many mites travel attached to the external elytral surface [95] (figure 2*p*). It was shown in the case of bark beetles that symbiotic microorganisms can affect several aspects of their development, ecology and behaviour [95].

### (i) Mating and courtship

Sexual dimorphism in elytral morphology and coloration can be found in many beetle lineages. In relatively rare cases, elytra are partially reduced in one sex (e.g. in males of *Myzomorphus* Tippmann (Cerambycidae)) or even lacking completely (e.g. in males of *Ozopemon* spp. (Curculionidae)). More subtle differences in elytral morphology between males and females can be found in many groups of Coleoptera. A well-known case are diving beetles of the genus *Dytiscus* Linnaeus (Dytiscidae), where the elytra are smooth in males but longitudinally grooved (figure 2*q*) in some but not all females [96]. For a long time it was assumed that the furrows increase the grip of the elaborate tarsal suction cups of the males during mating [97]. However, recent studies indicated that the uneven elytral structure weakens the male's grip, which likely increases the female control on mating [98]. Another example are females of some Cyclocephalini (Scarabaeidae), characterized by a specific expansion of the lateral elytral edge, forming a shelf or flange. It was hypothesized that this is part of a pre-copulatory sexual isolation mechanism used to control mounting of males [99]. Sexually selected adaptations can also be found on male elytra. For example, remarkable glandular notches are present on the elytra of males of *Clavicollis fugiens* (Marseul) (Anthicidae) (figure 2*r*), where large amounts of cantharidin are sequestered. These secretions are then transferred to females during mating as a nuptial gift [56]. Hooked elytral tips in *Pteroptyx* Olivier (Lampyridae) are used to clamp the female during mating, allowing for forceful insemination [100].

Elytra can also play an important role in copulatory courtship behaviour. Males of some species lick, rub or stroke the female's elytra or even drum on their surface [101–103], which might potentially induce the female to accept the male's attempts at intromission. Elytra can also play a role in mate recognition, as their cuticular hydrocarbons can function as contact sex pheromones [104,105], or help in species recognition, preventing cross-species mating [105,106].

Interaction with external male genitalia is likely a marginal function of elytra, but apparently they can play a supportive, stabilizing role during copulation and intromission in species with a strongly elongated penis. Such a phenomenon has been observed in the genus *Stenomastigus* Leleup (Scydmaeninae), characterized by sexually dimorphic elytra and an extremely long aedeagus [107]. Another example is the rove beetle *Aleochara tristis* Gravenhorst (Aleocharinae), where an extremely long aedeagus is secured between the pronotum and the elytral shoulder of the female. This mechanism allows its stepwise retraction, and subsequently the proper storage in the internal genital space of the males [108].

### (j) Acoustic communication

Elytra constitute an integral part of different types of stridulatory devices. The elytron usually plays a role of vibrating ‘file’, generating sound when stroked by other body parts. The elytral ‘file’ often constitutes an elevated and striated projection called ‘carina’ (figure 2*s*), or a set of small pointed tubercles. However, it might adopt a different form and vary in size and location. For example, in the elytro-femoral type of stridulatory device of the weevil *Erodiscus proximus* (Viana), the elytral ‘file’ constitutes a set of grooved tubercles located on the external elytral side near the lateral margin [109]. By contrast, in bark beetles of the genus *Dendroctonus* (Erichson), the pars stridens of an elytro-tergal stridulatory organ is formed by numerous ridges located on the ventral side of the posterior elytral margin [110].

The function of stridulation in Coleoptera is taxon-specific and can be associated with both intra- and interspecific communication. Beetles can produce characteristic sounds during a wide array of different behavioural patterns, including courtship, aggression and deterring predators, as part of acoustic mimicry or to communicate with conspecifics in eusocial species [111,112].

### (k) Hind wing folding

Closing of the wing cases along with abdominal pushing movements enables proper hind wing folding in beetles [113]. Moreover, the presence of small, cuticular protuberances (microtrichia) on elytra, hind wings and the abdominal surface (figure 2*t*) enables interlocking of the hind wings in their resting position. Microtrichia are grouped into specific fields and differ in terms of composition and direction of spicules, forming a kind of ‘zipper locking device’ [114]. It has also been shown that these microstructures enable complex wing folding patterns just by simple up-and-down movements [113].

## 5. Conclusion

The formation of rigid elytra took place in the Late Carboniferous and was likely linked with the exploration of new microhabitats, narrow spaces under bark of arborescent plants. A benefit of this ecological shift was reduced predation and competition, and additionally a stable environment with a constant high humidity.

A tent-like configuration with incompletely sclerotized forewings is part of the groundplan of Coleoptera in the widest sense [14]. This ancestral configuration was followed by elytral shortening and narrowing, increased sclerotization, a parallel arrangement of longitudinal veins, and the formation of inward directed epipleura [14]. The evolutionary transformations were completed in the Middle to Late Permian, with evenly sclerotized elytra and the formation of a secluded sub-elytral space. The elytral transformations including a gradual process of progressive sclerotization were enabled by a multi-step integration of body exoskeletalization genes into the wing gene network [8,35,37].

Even though the primary function of beetle elytra is apparently mechanical protection (e.g. [6]), closer scrutiny reveals that the modified forewings are in fact multi-functional organs. Aside from shielding the dorsal surface of the posterior body and internal organs from mechanical damage and predators, elytra can play a role in many functional contexts. This includes camouflage, mimicry, chemical defence, courtship and mating, flying, water retention and harvesting, body thermoregulation, or swimming and diving.

Numerous modifications of elytra occur, for instance the formation of spines or conspicuous colour patterns, or also different degrees of rigid connection or fusion. Partly reduced elytra occur in distantly related groups of Coleoptera,

It appears likely that the potential of the elytra to take over multiple tasks has contributed significantly to the unparalleled diversification of beetles.

## Data Availability

This article has no additional data.
